# *Echinacea* biotechnology: advances, commercialization and future considerations

**DOI:** 10.1080/13880209.2018.1501583

**Published:** 2018-10-10

**Authors:** Jessica L. Parsons, Stewart I. Cameron, Cory S. Harris, Myron L. Smith

**Affiliations:** a Ottawa-Carleton Institute of Biology, Ottawa, ON, Canada;; b Department of Biology, Carleton University, Ottawa, ON, Canada;; c Wood Science and Technology Centre, Hugh John Flemming Forestry Centre, Fredericton, NB, Canada;; d Department of Biology, University of Ottawa, Ottawa, ON, Canada

**Keywords:** Coneflowers, secondary metabolites, tissue culture, genetic transformation, elicitors

## Abstract

**Context:** Plants of the genus *Echinacea* (Asteraceae) are among the most popular herbal supplements on the market today. Recent studies indicate there are potential new applications and emerging markets for this natural health product (NHP).

**Objective:** This review aims to synthesize recent developments in *Echinacea* biotechnology and to identify promising applications for these advances in the industry.

**Methods:** A comprehensive survey of peer-reviewed publications was carried out, focusing on *Echinacea* biotechnology and impacts on phytochemistry. This article primarily covers research findings since 2007 and builds on earlier reviews on the biotechnology of *Echinacea*.

**Results:** Bioreactors, genetic engineering and controlled biotic or abiotic elicitation have the potential to significantly improve the yield, consistency and overall quality of *Echinacea* products. Using these technologies, a variety of new applications for *Echinacea* can be realized, such as the use of seed oil and antimicrobial and immune boosting feed additives for livestock.

**Conclusions:** New applications can take advantage of the well-established popularity of *Echinacea* as a NHP. *Echinacea* presents a myriad of potential health benefits, including anti-inflammatory, anxiolytic and antibiotic activities that have yet to be fully translated into new applications. The distinct chemistry and bioactivity of different *Echinacea* species and organs, moreover, can lead to interesting and diverse commercial opportunities.

## Introduction

Biotechnology centred on species of *Echinacea* Moench (Asteraceae) has grown substantially in recent decades, owing to the popularity of *Echinacea* as a natural health product (NHP). Originating in North America and part of the traditional pharmacopeia of Indigenous Peoples (Moerman 1998), *Echinacea* is now cultivated around the world and has an annual global market value estimated at approximately $1.3 billion (Blumenthal et al. 2003). Despite alternative taxonomies based on molecular, morphometric and phytochemical variation (Binns et al. 2002a), the traditional taxonomy of McGregor (1968) is still widely used and, recently supported by chloroplast genome data (Zhang et al. 2017), recognizes nine species within the genus. Commercial *Echinacea* preparations contain one or as many as three different species: *E. purpurea* (L.) Moench*, E. angustifolia* DC., and less frequently, *E. pallida* (Nutt.) Nutt., with *E*. *purpurea* making up about 80% of commercial production. Other recognized taxa, *E. laevigata* (C. L. Boynton and Beadle) S. F. Blake, *E. atrorubens* Nutt., *E. paradoxa* Norton, *E. sanguinea* Nutt., *E. simulata* McGregor and *E. tennesseensis* (Beadle) J. K. Small, are far less abundant and rarely utilized compared to the commercial species (McKeown 1999). Whereas the commercial species have received extensive research attention, these other *Echinacea* taxa have received almost none.

**Figure 1.**
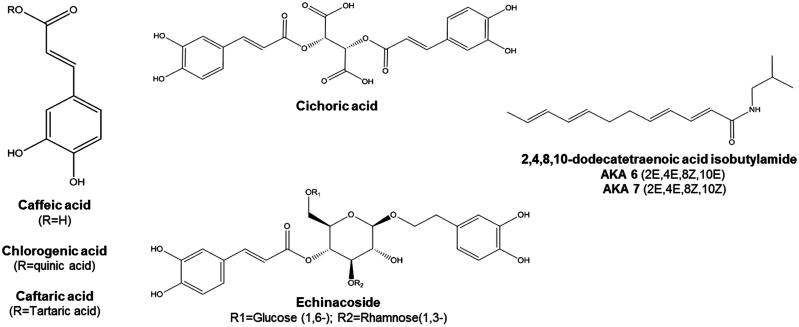
Structures of *Echinacea* phytochemicals with established bioactivity. Caffeic acid derivatives are represented by caffeic acid, chlorogenic acid, caftaric acid (left) as well as cichoric and echinacoside (middle). *Echinacea* alkylamides are represented by the isomers of 2,4,8,10-dodecatetraenoic acid isobutylamide; a diversity of alkylamides in *Echinacea* are similarly isobutylamides with alkyl chains of variable length and saturation.

Currently popular as an immune stimulant, *Echinacea* species were used by North American Indigenous Peoples as a treatment for throat infections, wounds and pain, and was historically used in Eclectic medicine for septic conditions (Shemluck 1982). Related pharmacological activities and therapeutic uses continue to be explored, including anti-inflammatory, analgesic, anxiolytic and antimicrobial activities (Hostettmann 2003; Abbasi et al. 2007a; Haller et al. 2013; Cruz et al. 2014; Shin et al. 2014). The main bioactive compounds present in *Echinacea* extracts are the phenolics, alkylamides and polysaccharide/glycoproteins (Figure 1). The phenolics include echinacoside, cynarin, cichoric acid, caftaric acid and chlorogenic acids (CADs), and possess antimicrobial and antioxidant activity. The alkylamides are a group of more than 30 lipophilic compounds with anti-inflammatory properties mediated through activation of the endocannabinoid system, exhibit antifungal properties and inhibit cyclooxygenase and lipoygenase enzyme activities. Polysaccharides/glycoproteins include complex carbohydrate moieties such as arabinogalactans that act as immunostimulants. Barnes et al. (2005) give a thorough inventory of bioactive compounds isolated from *Echinacea* and new activities continue to be reported and reviewed (Cruz et al. 2014; Murthy et al. 2014; Manayi et al. 2015).

**Figure 2.**
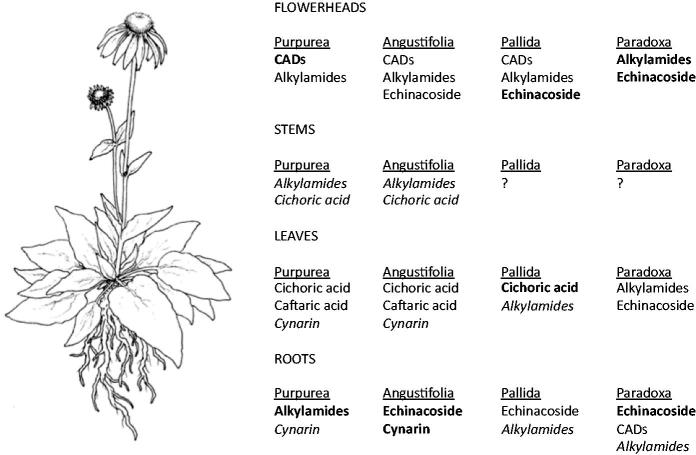
Localization of bioactive compounds in *E. purpurea, E. angustifolia, E. pallida,* and *E. paradoxa.* Compounds in bold are present at high concentrations; italics indicate compounds found in trace amount (Sloley et al. 2001; Binns et al. 2002b; Kabganian et al. 2003; Stuart and Wills 2003).

Differences in the composition and content of bioactive phytochemicals are inherent to *Echinacea* taxa (Figure 2). For example, *E. purpurea* roots completely lack echinacoside, a common constituent in the roots of other *Echinacea* species, but have very high levels of certain alkylamides, of which only trace amounts are found in the roots of other species (Sloley et al. 2001; Binns et al. 2002b; Murch et al. 2006). Typically, there can be significant variation in the phytochemistry of populations and/or individuals of the same species as well, particularly in *E. angustifolia,* for which there are established chemoraces (Kapteyn et al. 2002; Binns et al. 2002c; Liu et al. 2006; Chuang et al. 2010; Abbasi et al. 2012). Additionally, in all *Echinacea* species studied to date, the localization and content of active metabolites changes over time, both seasonally and with plant age, and varies between plant parts (Figure 2) (Choffe et al. 2000; Binns et al. 2002b). The most recent study on the localization of alkylamides in *E. purpurea* examined alkylamide content in a total of 36 tissues (Rizhsky et al. 2016), not including the seed. Particularly high concentrations of alkylamides were found in petals and disc flowers, and moderate concentrations were noted in receptacles of mature flower heads. Our group recently observed that the glands on the outer surface of *Echinacea* seeds (beneath the perianth) are also enriched in alkylamides (Parsons et al. 2018). *E. purpurea* and *E. angustifolia* roots and flower heads generally have the highest concentrations of bioactive compounds, whereas the leaves and stems have low concentrations of metabolites, and are rarely used in preparing NHPs (Kabganian et al. 2003; Qu et al. 2005; Chen et al. 2009). However, ascorbic acid (vitamin C) accumulates in the leaves, which could augment immune functions (Zagumennikov et al. 2015). This pattern of localization differs from *E. paradoxa*, where phytochemicals are concentrated more in the flower heads (Chen et al. 2009), and may differ in other rare species as well. This variation potentially provides a basis for selective breeding programs, selection of useful cultivars, and multiple product streams from different parts of the same plant.

Despite the success of *Echinacea* on the market, challenges such as fungal pathogens, seed dormancy, low germination rates and a relatively long maturation time still pose problems for the industry, with the biggest challenge being product standardization (Liu et al. 2006; Zheng et al. 2006; Abbasi et al. 2007a; Kindscher et al. 2008; Maggini et al. 2012). Different commercial brands use diverse genotypes, plant parts and blends as well as different growing conditions, harvesting times and extraction methods, leading to qualitative and quantitative inconsistencies in bioactive compounds composition (Stuart and Wills 2003; Abbasi et al. 2007a; Jones et al. 2009). As industry faces these challenges, research has focused on finding ways to meet a strong demand for consistent, high-quality *Echinacea* products. In this article, we review the most promising developments and trends in *Echinacea* biotechnology, focusing on propagation, standardization and optimization of both the production process and the quality of plant material. We will examine advantages and disadvantages of the various technologies, and highlight future applications focusing on diversification and sustainability of the *Echinacea* industry.

## *Echinacea* production in bioreactors

Traditionally, *Echinacea* has been propagated by seed, crown division or root cuttings but these processes are time-consuming, have limited propagation potential for producing large numbers of plants and can produce genetically variable plant material. Tissue culture methods – growing plants *in vitro* – have the potential to solve a number of the problems related to *Echinacea* propagation, including cultivation bottlenecks, the use of rare species and product standardization. Owing to the rapid development of tissue culture technology, a large – yet fragmented – body of research has accumulated on its application in *Echinacea*.

*In vitro* micropropagation techniques, such as adventitious root and shoot culture, and somatic embryogenesis, can produce hundreds of clonal plants from cuttings of a parent plant. Abbasi et al. (2007a) provide a thorough review of micropropagation techniques in *Echinacea*, all of which allow for more consistent secondary metabolite profiles associated with isogenic lines, year round cultivation and reduction of microbial contamination. Micropropagation can also be used to create plants having unique phytochemical profiles by culturing different parts of a parent plant. For example, shoots regenerated from *E. angustifolia* flower stalks have proportionately higher content of CADs compared to shoots generated from leaf explants (Lucchesini et al. 2009). Somatic embryogenesis by tissue culture, and to a lesser extent organogenesis, can induce genetic changes (Chuang et al. 2009) – a phenomenon called somaclonal variation. Micropropagation has been accomplished with *E. purpurea, E. pallida*, *E. angustifolia* and *E. tennesseensis* where, as expected, clonal plants have similar phytochemical profiles, showing only minor somaclonal variation (Abbasi et al. 2007a; Moraes et al. 2011; Butiuc-Keul et al. 2012 for additional studies). Although micropropagation provides a rapid way to generate plants, the process is still time consuming and labour intensive. These limitations are likely why, despite the popularity of herbal medicines, commercial production of *Echinacea* rarely employs cell culture techniques (Baque et al. 2012). In order to make tissue culture methods viable at industrial scales, bioreactors are considered an alternative culturing strategy. In addition to simpler technologies, one of the newer strategies is to use temporary immersion systems (TISs), where tissues are briefly bathed in nutrient medium then drained at specified intervals daily. Such bioreactor systems are modular and can mass produce clonal materials in the range of hundreds to tens of thousands of plants, making them suitable for use in genetic improvement programs.

The simplest bioreactors are used mainly for cell suspension cultures. However, plant cell cultures may not provide a complete phytochemical profile since some compounds are only produced in differentiated cells or following environmental cues. To deal with these limitations, several different bioreactor systems have been developed, including gas-phase, TISs, and hybrid bioreactors, all of which give cultures improved access to air and allow for the growth of differentiated organ cultures (Georgiev et al. 2014). For example, the recently developed “Plantform” bioreactor allowed *E. purpurea* tissue cultures to produce more adventitious shoots and a greater total biomass compared to cultures grown on solid media (Welander et al. 2014). These bioreactors use automated systems that provide a sterile environment, and produce plants that can be ready for harvest in just a few months. In an airlift bioreactor, both *E. purpurea* and *E. angustifolia* adventitious root cultures consistently produce up to 10 times the biomass of field grown plants after 5 weeks, and higher levels of active phytochemicals, including CADs, echinacoside and cynarin (Wu et al. 2007a; Jeong et al. 2009; Baque et al. 2012; Cui et al. 2013). Depending on the species used, co-culturing adventitious roots of different *Echinacea* species in balloon-type airlift bioreactors can also increase the production of biomass and bioactives, including the synthesis of metabolites absent from single-species cultures (Wu et al. 2017). Whether the increase in phytochemical content is due to stress on the plant cells in the *in vitro* environment, the availability of excess nutrients in the medium, or to other aspects of the culture environment, remains unclear.

Among the most important limitations of bioreactors is their capacity for scale up and cost. Biomass production often decreases at larger scales, so strategies such as medium replenishment are employed to improve biomass production and phytochemical content in cultured roots (Wu et al. 2007b). Although culture vessels of up to 75,000 L have been used for suspension culture (Ruffoni et al. 2010), scale-up tests have yet to be carried out with new TIS bioreactors. To date, *E. purpurea* and *E. angustifolia* adventitious roots have been cultured in balloon-type bubble bioreactors of up to 500 L and in drum-type bioreactors of up to 1000 L without noticeable adverse effects on growth (Wu et al. 2007a; Ruffoni et al. 2010). Nonetheless, commercial use of bioreactors remains costly and is limited to production of cosmetics and high-value pharmaceuticals such as paclitaxel. Although not yet routinely used in the *Echinacea* industry, bioreactors are becoming the standard when performing tissue culture at an experimental scale. Bioreactors offer a means of producing standardized plant material at a scale unmatched by field production, and the contained nature of bioreactors allows for the use of specialized media, conditions, elicitors, growth enhancers and year-round production. With a viable and consistent propagation method in hand, the focus now shifts to improving production efficiency, economy and the quality of plant material, in terms of both biomass and phytochemical content.

## Genetic improvement

Conventional selective breeding techniques have traditionally led to the gradual improvement of many plant species. While industry will undoubtedly continue to develop “improved” varieties, published *Echinacea* breeding studies (and patents) have focused primarily on ornamentals (Ault 2002; Korlipara 2008) and reducing seed dormancy (Qu and Widrlechner 2012). Traditional selective breeding of *Echinacea* can make use of the existing genetic and phenotypic variation in commercial and wild collected plants and is widely accepted by the public, including within the organic farming industry. Conversely, direct alteration of the genome of a plant through molecular genetic techniques is the most precise way to modify developmental and biosynthetic processes. Whereas public concerns will likely continue to impede the use of Genetically Modified Organisms (GMOs), several potentially “organically acceptable” biotechnological approaches have been developed to modify *Echinacea*, including transformation with *Agrobacterium* and the induction of polyploids.

Hairy root culture utilizes the natural ability of the soil bacterium *Rhizobium rhizogenes* (formerly *Agrobacterium rhizogenes*) to infect and transform plant tissue. The bacterial Ri plasmid is transferred into the plant genome causing neoplastic outgrowths, but incorporation of a set of genes, *rolA*, *rolB* and *rolC*, causes roots to grow from the infected site instead of an undifferentiated cell mass (Nilsson and Olsson 1997; Pistelli et al. 2010). Hairy root cultures have several properties that are useful for research and industry, including accelerated growth, spontaneous regeneration of shoots, as well as chemical and morphological similarity to the roots of a wild-type plant (Tepfer 1990; Guillon et al. 2006). Hairy root cultures of all three commercially important *Echinacea* species produce high levels of secondary metabolites, including polysaccharides, alkylamides, CADs and other phenolics (Trypsteen et al. 1991; Liu et al. 2006; Wang et al. 2006; Romero et al. 2009; Pistelli et al. 2010). Transformed roots are genetically stable, and maintain a constant production of metabolites over a long period of time (Wu et al. 2006). The rapid growth of hairy root cultures on hormone-free media makes them an excellent way to generate biomass quickly, or to clonally propagate plants.

The discovery of *R. rhizogenes*-based hairy root transformation systems in higher plants provides other opportunities to engineer useful traits in *Echinacea*. Again, public acceptance of GMOs may limit the application of this useful technology. As an example, glufosinate-resistance and a fungal resistance chitinase gene were simultaneously transferred into *E. purpurea* using *R. tumefaciens* (Hanafy et al. 2010). Considering *Echinacea* plants in the field are particularly susceptible to weed competition and fungal pathogens, this study represents a useful demonstration model. Several factors are noted to influence *Rhizabium*-based transformation efficiency of *Echinacea*, and there is room for optimization. For example, the efficacy of the utilized bacterial strain is important; A4 strains were superior for transforming *Echinacea* leaf explants, whereas R1000 strains worked best with petioles (Wang et al. 2006). Overall, early development stages, such as cotyledon tissue, are more easily transformed and sonication is up to twice as effective for producing transformants compared to the traditional methods of wounding with a sterile needle to enhance *R. rhizogenes*-mediated gene transfer (Kumar et al. 2006). Addition of inducers to the medium during co-cultivation of agrobacterium with the plant tissue also improves efficacy. For example, indole-3-butyric acid (IBA) increases production of hairy roots in *Echinacea* by as much as 14 times (Romero et al. 2009). Other inducers of *Rhizobium*-associated gene transfer in plants (e.g., 6-benzylaminopurine, 2,4-dichlorophenoxyacetic acid) have been applied to *Echinacea* hairy root cultures to improve transformation but their effectiveness relative to no treatment has not been investigated empirically (Trypsteen et al. 1991; Wang et al. 2006*)*.

Likewise, the manipulation of ploidy can cause changes to morphology and phytochemical content of plants. Naturally occurring polyploids are commonly used in agriculture and widely accepted by the public. Triploid, tetraploid and hexaploid *Echinacea* plants have been developed, with tetraploids (4× = 44) being the best studied. In comparison to wild-type diploids, the tetraploids studied have altered leaf, root, and flower morphology, reduced seed set and dwarfed phenotypes (Nilanthi et al. 2009; Koul et al. 2010; Abdoli et al. 2013; Xu et al. 2014; Chen et al. 2016). Tetraploid plants have similar phytochemical profiles to wild types, but they consistently yield higher levels of CADs, particularly in the leaves. Increased alkylamide content in the leaves and roots of tetraploids has also been noted (Koul et al. 2010; Abdoli et al. 2013; Xu et al. 2014). The reduced biomass production due to polyploid dwarf phenotypes currently makes ploidy manipulation an impractical way to improve the quality of *Echinacea* plant material. However, supplementing culture media with 0.3 mg/L IBA can accelerate the emergence of roots and the increased rooting rate of tetraploid shoots *in vitro* whereas IBA has no positive effect on cultured haploid or diploid shoots (Chen et al. 2016).

## Elicitation of secondary metabolites to produce high quality plant material

Bioreactors and micropropagation techniques allow a large number of plants to be produced in a short period of time but developing biomass with a higher concentration of bioactive compounds ultimately makes for a more efficient industrial process. Several methods to enhance the production of secondary metabolites, other than genetic engineering, are currently being studied. In particular, the use of elicitors (e.g., plant hormones, stress signaling molecules and both biotic and abiotic compounds, as well as physical injury) show promise in stimulating the production of bioactive secondary metabolites (reviewed in Abbasi et al. 2007a and updated in Table 1). In general, the basis of elicitation is the activation of a plant’s defence response, which up-regulates the production of many bioactive compounds of commercial and industrial value. Elicitation involves either the direct addition of signal compounds implicated in the stress response or applying compounds that cause tissues to produce stress signals endogenously. For example, nitric oxide (NO) is an important signalling molecule in the plant defence response. In adventitious root cultures of *E. purpurea,* adding sodium nitroprusside (an exogenous NO producer) to the growth medium increased the accumulation of flavonoids and CADs (Wu et al. 2007c). Natural stress mediators can also be applied as a foliar spray to a mature plant. Salicylic acid elicits a twofold increase in cichoric and caftaric acid in *E. purpurea* flower heads, and an almost fourfold increase of CAD in the roots when applied as a foliar spray to field-grown plants (Kuzel et al. 2009). Elicitors such as yeast extract stimulate the production of phenolics in *Echinacea*, presumably by mimicking a pathogenic fungal infection (Li and Barz 2006). From Table 1, it should be noted that the majority of recent elicitor research has been carried out with *E. purpurea* (15/19 studies), *E. angustifolia* (4/19) and *E. pallida* (2/19). Comprehensive screening studies that include other taxa are warranted for biotechnology potential since it is clear that most of the studies report increased metabolite production following administration of elicitors. In particular, CADs, but also phenolics and flavonoids appear to be most responsive to elicitor induction, likely through phenylalanine ammonia lyase (PAL) up-regulation and defence response. Relatively few studies state whether or not other metabolites such as echinacoside, alkylamides or polysaccharides are similarly increased by application of elicitors, so the full potential of inducing secondary metabolite production remains unknown.

**Table 1.** Review of elicitor compounds tested in *Echinacea* to enhance secondary metabolite content.

Type	Elicitor	Concentration used	Variety	Effects	Reference
Abiotic	Titanium (IV) ascorbate	Foliar spray – 10 μM	PPA	Increased caftaric, chlorogenic acid in roots, and increased root mass	Kuzel et al. 2009
Biotic	Yeast extract elicitor	1mg/mL	PPA	Increased production of phenolics, including novel phenolics	Li and Barz 2006
Growth Regulator	Dimethyl amino hexanoate (with 0.3 mg/L IBA)	0.08 mg/L for petioles, 0.16 mg/L for leaves, 0.01 mg/L for roots	PPA	Enhanced shoot regeneration, increased plant growth and final biomass	Chen et al. 2013
	Gibberellic acid	0.025 μM	PPA	Thicker roots, increased overall biomass, increased levels of anthocyanins and cichoric acid	Abbasi et al. 2012
		150 μM	PPA	Increase in caftaric acid in early culture stage	Jones et al. 2009
	Indole-3-butyric acid (IBA)	1 mg/mL	PLL	Induces root regeneration, increases CAD content	Wu et al. 2012
		2 mg/L	ANG	Increased final weight of root cultures, increased total phenolics and flavanoids	Wu et al. 2006
		15 μM	PPA	increased levels of cichoric and caftaric acid, and detectable levels of echinacoside	Murch et al. 2006
Herbicide	Glyphosate treatment + l-tryptophan feeding	0.5 mM glyphosate, 0.5 mM l-tryptophan	PPA	Higher levels of CADs, phenols, and flavanoids	Mobin et al. 2015
	Palcobutrazole + Gibberellic acid	100 μM Palcobutrazol, 50 μM GA	PPA	Increased caftaric and cichoric acid in roots, slight drop in shoots	Jones et al. 2009
Stress Response Molecule	Acetylsalicylic acid	Foliar spray – 10 μM	PPA	Increased caftaric acid in tops	Kuzel et al. 2009
	Jasmonic acid	10–40 μM	PPA, ANG, PLL	Several fold increase in alkylamides, but decreased root growth	Romero et al. 2009
	Methyl Jasmonate	5 μM	PPA	Increased production of phenolics, including novel phenolics	Li and Barz 2006
	Methyl Jasmonate, Methyl salicylate	200 μM	ANG	Increase of of echinacoside and cichoric acid	Baque et al. 2012
		100 μM	ANG	Increased polyphenolics, flavanoids, and CADs in root culture	Cui et al. 2013
		Foliar spray – 10 μM	PPA	Increased caftaric acid in tops, cichoric and chlorogenic acid in roots, and mass of roots	Kuzel et al. 2009
	Salycilic acid	Foliar spray – 10 μM for tops, 1000 μM for roots	PPA	Increased cichoric and caftaric acid in tops, and cichoric acid, and mass in roots	Kuzel et al. 2009
	Sodium nitroprusside	100 μM	PPA	Increased phenolics, flavanoids, CADS, and antioxidant potential	Wu et al. 2007c

E. purpurea: PPA; E. angustifolia: ANG; E. pallida: PLL.

Despite these impressive effects on phytochemical content, elicitors can also have an inhibitory effect on growth (Baque et al. 2012). As such, a two-phased approach – adding elicitors only after the cell culture or plant has had time to grow – may be necessary in order to optimize phytochemical production. For example, by adding methyl jasmonate to the medium on day 28, Cui et al. (2013) increased the echinacoside content in root cultures of *E. angustifolia* threefold without reducing the biomass. The use of chemical elicitors is particularly important in industrial-scale bioreactors since the production of both biomass and phytochemicals often decreases at large scales (Wu et al. 2007b). Additional tests on the application of elicitors to field grown plants would also be beneficial, since effects may differ under the diversity of field conditions.

Commercial use of elicitors needs to balance secondary product yields against cost and the potential residual toxicity of an elicitor remaining in the harvested tissue. In addition, since *Echinacea* is sold and regulated as a NHP or supplement (in North America), and since there is a consumer preference for naturally grown products, consideration should be given to the type of elicitor used; growth hormones and stress mediators may be preferred over abiotic elicitors and herbicides.

## Other approaches to optimizing secondary metabolite production

In addition to genetic transformation and elicitor treatments, a number of other factors can affect the production of secondary metabolites in *Echinacea* culture. Aspects of the growing environment, such as growth medium and light regime, as well as abiotic treatments like ultrasound or elevated UV A/B exposure that cause physical cellular damage, have been considered for the optimization of *Echinacea* products. These alternative elicitation methods have the advantage of leaving no residues but have only been tested in *E. purpurea* and need to be evaluated in other species for their commercialization potential.

Growth medium is the basis of tissue culture, and numerous studies have assessed what combination of nutrients produce the best growing environment for *Echinacea* cultures. Depending on the *Echinacea* species and tissue being cultured, the optimal medium may differ. For instance, Wu et al. (2007a), found that the maximum biomass of adventitious roots of *E. purpurea* could be obtained on one-quarter strength Murashige Skoog (MS) medium with 50 g/L sucrose and 1 mg/L IBA, but in earlier work with root cultures *in E. angustifolia,* half strength MS produced roots with more biomass and a higher content of phenolic compounds than one-quarter strength MS (Wu et al. 2006). Focusing on alkylamide production in hairy roots, Romero et al. (2009) reported that half-strength Gamborg’s B5 medium was best for maintaining hairy root production and that the addition of IBA increased growth rate by 14-fold with no impact on alkylamide production, which was further elevated in the presence of the elicitor, jasmonic acid.

Ultrasound treatment is a recently developed method of increasing plant secondary metabolite content, and as such, has not been extensively tested with *Echinacea*. Two studies with *E. purpurea* hairy roots grown in bioreactors found that one 6 minute ultrasound treatment at 40 kHz between days 15 and 20 of culture significantly increased both fresh weight and cichoric acid content over 30 days (Abbasi et al. 2009; Liu et al. 2012). Although the exact mechanism is unknown, the effects of ultrasound appear linked to an increase in PAL activity. Both studies noted an increase in cichoric acid, with one also reporting significant increases in anthocyanins and lignins (Abbasi et al. 2009), the biosynthesis of which are all linked to PAL enzyme activity. Liu et al. (2012) observed enhanced CAD biosynthesis related to increases in both *rolB*-regulated auxin biosynthesis and PAL activity. Accordingly, ultrasound treatment may be less effective at eliciting secondary metabolism using culturing techniques other than hairy roots. Altered secondary metabolism may also result from ultrasound-induced physical damage to the cells, causing a general stress response. Indeed, Liu et al. (2012) noted that stimulating hairy roots with ultrasound caused a significant decrease in biomass accumulation, likely indicating cell damage or stress. This method of elicitation is very simple, requires no chemical input, and leaves no residues, and therefore merits study with other forms of tissue culture as well as with other species of *Echinacea*.

Light is essential for the growth of plants and for the regeneration of shoots in culture. While callus, root and suspension cultures are generally maintained in the dark, exposing these cultures to light can have beneficial effects on their phytochemical content. Continuous light for 14 days significantly increased CAD levels in cell suspensions of *E. angustifolia* (Guarnerio et al. 2012). Similarly, hairy root cultures of *E. purpurea* incubated under continuous light showed not only an increase in CADs but also thicker roots that developed a purple colour, indicating the production of anthocyanins. Increased CAD and anthocyanin production have been linked to the activation of PAL enzymes, although the mechanism of PAL activation by light is unknown (Abbasi et al. 2007b). Enhanced production of CADs and anthocyanins was also observed by Abbasi et al. (2012) with the application of gibberellic acid to *E. purpurea* hairy roots. If light treatment can produce effects comparable to certain elicitors, then light-induced effects on secondary metabolite production warrants further investigation. In particular, it would be interesting to test different wavelengths, intensities and light/dark regimes to determine if the same or greater effects can be achieved without continuous bright light.

As has been demonstrated with other species, an extension of abiotic light treatment is the recently reported application of elevated ultraviolet light during *Echinacea* callus and cell culture to alter secondary compound formation. Manaf et al. (2016) tested the effects of UV-B radiation on *E. purpurea* cultures for short periods in varying exposures. The effects were variable, depending on the dose–time response. All UV-B treatments increased caffeic acid and antioxidant activity of callus cells and growth parameters, total phenols content and antioxidant activity of cell suspensions in a dose-dependent manner. The same group also tested both types of *Echinacea* cultures with varying doses of UV-C with similar results (Abd El-Aal et al. 2016).

Endophytes, microbial species that colonize plants without causing disease symptoms, are associated with almost all plants on Earth. Endophytes can be isolated from all parts of field-grown *Echinacea*, including the seeds, leaves, stems and roots. The most common fungal genera in the roots of *Echinacea* include *Glomus*, *Cladosporium*, *Alternaria* and *Fusarium* (Lata et al. 2006; Araim et al. 2009; Zubek and Błaszkowski 2009; Rosa et al. 2012; Moszczyńska et al. 2013). Endophytes form symbiotic relationships with the plant, using photosynthesized sugars for nutrition, in turn helping the plant to uptake nutrients (particularly nitrogen), and defend against herbivores and pathogenic microbes (Arnold and Lutzoni 2007; Aly et al. 2011). Plants colonized with endophytic fungi are less often infected by pathogens, show increased growth rates, and have improved stress tolerance (Saikkonen et al. 1998; Lata et al. 2006; Araim et al. 2009; Zubek and Błaszkowski 2009; Gualandi et al. 2014). Notably, colonization with arbuscular mycorrhizal fungi impacts phytochemical content, increasing CADs in the roots of *E. purpurea* (Araim et al. 2009). The mechanism of this effect in *Echinacea* is unknown, and could potentially be attributed to increased uptake of nutrients, elicitation of the plant’s defense response (including PAL up-regulation), or production of bioactive compounds by the endophytes, among other explanations. Nevertheless, manipulation of field grown plants to encourage specific endophytes may increase yields of secondary metabolites.

## Future directions in *Echinacea* research and industry

The development of new biotechnologies provides many options for improving *Echinacea* NHPs and other products. Moving forward, the benefits and drawbacks of each of these approaches should be considered in terms of improvements in yield, optimization and standardization of phytochemistry, propagation efficiency, cost, public perception and ease of use at scale.

As previously noted, bioreactors allow for the rapid growth of cultures and produce cloned propagules, ensuring consistent phytochemical content. *In vitro* culturing reduces contamination by plant pathogens and other microbes and is less labour intensive than field cultivation. However, individual plant tissues may not produce a full range of phytochemicals, and biomass production may be limited by culture techniques and equipment. Despite reduced labour, the cost of bioreactor culture is high, as it requires specialized materials, facilities and personnel training.

Genetic engineering has the potential to improve plant material in several ways but has multiple drawbacks making it currently impractical for use in the *Echinacea* industry. Genetic transformation may not improve propagation efficiency, does not necessarily increase yield (except hairy roots), and does not guarantee more standardized plant material. Most importantly, the NHP market may not readily accept the use of GMOs. Despite the fact that ploidy variation and hairy root disease occur naturally, market research should precede employment of such technologies. Genetic transformation is useful to study the growth and biochemistry of plants but, since elicitors and selective breeding can produce similar improvements in yield and quality, genetic engineering may not be the best option for industry.

Elicitors are easy to use, do not change propagation efficiency, and can be applied to either organ cultures or field-grown plants. Elicitors effectively improve phytochemical content and some, such as IBA, can increase yield. Other optimization techniques such as ultrasound, UV and ozone (abiotic elicitors) can similarly produce increases in phytochemical content in tissue culture. Administering ultrasound is inexpensive, simple to use, and will not result in toxic residues, but improper use may lead to decreases in yield and ultrasound technologies have not yet been adapted for use with field cultivation.

In general, bioreactors may have advantages for propagating cultivars through rapid production of cloned propagules. However, a focus on the growth of whole plants for the production of herbal medicines seems the most beneficial overall currently. Whole plants are technically less complicated to maintain and can produce more complete phytochemical profiles. Even though the price of tissue culture has come down, it is still not feasible to grow full plants to maturity at industrial scale using bioreactors. Therefore, it may be best to use tissue culture as a method of propagation followed by growing cloned plantlets in more traditional field, greenhouse or hydroponic systems. Chemical elicitors are most effective for increasing phytochemical content, and can be included in the production process, along with control of light regimes during indoor production. Such models would take advantage of both the benefits of tissue culture and the existing cultivation space.

Growing entire plants would also be worthwhile since each part of the plant has unique properties that can be used for different kinds of products. *Echinacea* supplements on the market today are most often an extract of the roots, flower heads or both, with the leaves of *E. pallida* occasionally included. Finding uses for the remaining plant parts and developing alternate applications of *Echinacea* is the logical next step for research and industry. There is very little information available on the phytochemical and medicinal properties of rare *Echinacea* species, however *in vitro* culturing technology may now allow for the growth and study of these species without disturbing natural populations. Non-commercial *Echinacea* species may yield compounds that are not present in *E. purpurea, E. angustifolia* or *E. pallida* (Binns et al. 2002b)

Emerging market opportunities for *Echinacea* include additives in animal feed. Studies on chickens, pigs, rainbow trout and horses have consistently found that *Echinacea* feed additives improve immune activity, including increases in lymphocytes, phagocytosis and globulin content (Williams and Lamprecht 2008; Böhmer et al. 2009; Grashorn 2010; Dehkordi et al. 2011; Oskoii et al. 2012). The addition of *Echinacea* to feed or water also improved the efficacy of vaccines for fowl influenza, swine erysipelas and Newcastle disease virus in fowl, increasing the antibody titers in livestock (Maass et al. 2005; Böhmer et al. 2009; Dehkordi et al. 2011; Najafzadeh et al. 2011). Reducing the incidence of infection in livestock improves growth rate and decreases the chances that pathogens will be transferred between animals or to humans. Restrictions on the addition of synthetic antibiotics to animal feed in several countries may soon expand the market for herbal medicines in the livestock industry. The only negative reports to date on *Echinacea* feed additives were minor allergic reactions in horses (Williams and Lamprecht 2008). Addition of *E. purpurea* to feed did not significantly alter growth characteristics of quail (Sahin et al. 2012). Nevertheless, residual *Echinacea* bi-products from human health applications should be explored as value-added animal feed supplements, including uses for leaves, stems and seed cobs. More research is needed in order to determine the most effective form, delivery method, and dosage for employing *Echinacea* as a commercial feed additive.

*Echinacea* seed also may have additional market potential. Only a fraction of the seed produced by *Echinacea* is required for traditional plant propagation. Expanded use of tissue culture also means that additional seed could be harvested solely for use in NHPs. Indeed, use of seeds may add value to *Echinacea* crops since seeds are generally not incorporated into commercial products. Seed oils from all three commercial *Echinacea* species are very nutritious, being high in oleic acid, palmitic acid, linoleic acid, vitamin E (28–85 mg/100 g oil) and other bioactive compounds (Oomah et al. 2006; Vandyshev et al. 2009; Parsons et al. 2018). Seed oil yields range from 13 to 23%, depending on species and seed size, with *E. purpurea* seeds generating the greatest volume and highest quality of oil. The seeds of *E. purpurea* and *E. angustifolia* contain 0.75 and 1.06 mg of bioactive alkylamides per gram, respectively (He et al. 1998). Oils from other members of the Asteraceae family, such as sunflower oil, are commonly used for both dietary and industrial purposes.

*Echinacea* essential oil also contains a number of medicinal compounds including germacrene-D, a sesquiterpine hydrocarbon with antimicrobial properties, and alkylamides, which can be detected by the tingling sensation caused on the tongue when the oil is tasted (Mirjalili et al. 2006; Oomah et al. 2006). *E. purpurea* essential oil significantly reduced inflammatory swelling in mice and rats, and decreased the levels of cytokines IL-2, IL-6 and TNF-α in the blood (Yu et al. 2013). These effects are also characteristic after administering alkylamides in animal models. With further research and the development of standard extraction procedures, *Echinacea* seed oil and essential oils also have the potential to become successful product lines within the *Echinacea* industry.

*Echinacea* is almost exclusively sold as an immune-boosting supplement in the contemporary US market. However, the development of micropropagation, bioreactors and elicitors make it possible to take advantage of other bioactivities. Extensive study demonstrates that *Echinacea* extracts have antibacterial, antiviral and antifungal activities (Merali et al. 2003; Sharma et al. 2008; Mir-Rashed et al. 2010; Hudson 2012). *Echinacea* alkylamides were shown to have an interesting mode of action through perturbing the fungal cell wall/membrane complex – an ideal antifungal target that is unique to fungi (Cruz et al. 2014). Of those tested, one structural class of alkylamides, the diynoic alkylamides, showed the greatest antifungal and cell wall disruption activities. The natural variability in the phytochemical profiles of *Echinacea* plants, combined with high-throughput screening and use of elicitors make it possible to quickly select and propagate various cultivars with unique metabolic profiles, tailored to specific functions such as production of antifungals. Alternatively, *Echinacea* varieties could be selected for large seeds with nutritious seed oil, or modified to produce more CAD in the flowers, which could be used as a dietary supplement. The roots and flowers of cultivars rich in alkylamides and cichoric acid could be used to make antibacterial face washes, shampoos or creams. Like other members of the Asteraceae family, *Echinacea* contains some polyacetylene compounds that are phototoxic, but these are unstable and could be inactivated by minimal processing (Chen et al. 2010; Chen et al. 2013). *Echinacea* leaves, high in vitamin C and phenolic metabolites, could also be marketed as a NHP. This potential diversity of uses has yet to be harnessed by the industry.

Moving forward, a combination of tissue culture, chemical treatments and traditional field cultivation will likely be used to generate a new higher standard of production and phytochemical quality. These advances will provide the opportunity to establish a greater variety of *Echinacea* products to keep up with ever-expanding market opportunities.
